# Chewing ability in an urban and rural population over 40 years in Shandong Province, China

**DOI:** 10.1007/s00784-012-0822-1

**Published:** 2012-09-02

**Authors:** Qian Zhang, Dick J. Witter, Ewald M. Bronkhorst, Nico H. J. Creugers

**Affiliations:** 1 Department of Prosthetic Dentistry, Affiliated Hospital of Medical School, Qingdao University, Jiangsu Road 16#, Qingdao, People’s Republic of China; 2 Department of Oral Function and Prosthetic Dentistry, College of Dental Science, Radboud University Nijmegen Medical Centre, Philips van Leydenlaan 25, 6525 EX Nijmegen, The Netherlands; 3 Department of Preventive and Restorative Dentistry, College of Dental Science, Radboud University Nijmegen Medical Centre, Philips van Leydenlaan 25, 6525 EX Nijmegen, The Netherlands

**Keywords:** Chewing ability, Occlusal status, Hierarchical dental functional classification system, Chinese adults

## Abstract

**Objectives:**

This study aimed to assess chewing ability related to dental status.

**Material and methods:**

One thousand four hundred sixty-two Chinese subjects over 40 years, dentate in both jaws, were categorized in a hierarchical functional classification system with and without tooth replacements. Chewing ability was analyzed using multivariable logistic regression including five dental conditions (≥10 teeth in each jaw’; ‘complete anterior regions’; “sufficient premolar regions’ (≥3 posterior occluding pairs (POPs)); ‘sufficient molar regions’ (bilaterally ≥1 POP); and tooth replacement), adjusted for six background variables. Likelihood ratios for chewing problems were assessed at each level of the hierarchical classification system based on these dental conditions.

**Results:**

Seventy-eight to 91 % of subjects reported no or minor chewing problems. The conditions ‘≥10 teeth in each jaw’, and ‘complete anterior regions’ were not associated, whereas ‘sufficient’ premolar regions’ and ‘sufficient molar regions’ were associated with chewing problems (Ors, 0.33–0.58). If classified hierarchically, the condition ‘≥10 teeth in each jaw’ was relevant for chewing problems (likelihood ratios 3.3–3.7). ‘Sufficient premolar region’ and ‘sufficient molar region’ were relevant to reduce the likelihood ratios for having chewing problems (both approximately with a factor 2), both for soft and for hard foods. Subjects with artificial teeth added had similar chance for chewing problems compared to counterparts with natural teeth only. However, if comparing replaced teeth with natural teeth, subjects with tooth replacement showed higher chance for chewing problems.

**Conclusions:**

Chewing ability was strongly associated with dental conditions.

**Clinical relevance:**

The presence of at least 10 teeth in each jaw had highest impact on chewing ability.

## Introduction

One of the most immediate and important functional consequences of tooth loss is a reduction in chewing ability. Oral rehabilitation might restore partially the objective chewing capacity and might lead to an increased appreciation of chewing ability. The relationship among chewing ability and the state of dentition has been subject of numerous studies, of which the majority of studies report a strong relationship [1–4]. Other studies, however, are less clear about a direct relationship and reported ambiguous findings regarding the number and location of teeth needed for a satisfactory chewing function [5, 6]. People’s satisfaction with chewing ability is not determined entirely by their mechanical chewing function. Instead, it is a complex measure that embraces broad physical, social, and psychological components [7]. It has been stated that the chewing process is an individually determined and adaptive process [8, 9]. For instance, in elderly populations chewing ability has been associated with several non-dental functional impairments, such as decreased biting force and reduced saliva flow, as well as with factors such as general health and psychological and social well-being [10–13]. Also, recent studies have indicated that impaired chewing ability affects oral health-related quality of life (OHRQoL) [6, 14]. However, it has also been reported that the correlation between perceived chewing ability and OHRQoL is not substantially influenced by number of teeth and age, but by gender, level of education, treatment demand, and prosthodontic status [2, 6].

A systematic review based on DMFT data reported that the number of missing teeth in Chinese adults increases with age from approximately 2 at the age of 40 years to approximately 12 at the age of 65 years [15]. Information about the relationship of number of teeth and chewing ability for Chinese people is scarce. Zeng et al. [16] have described the effect of clinical dental status on chewing ability for a Chinese population in Guangxi. In that study decreased chewing ability was significantly related with having fewer (occluding) teeth, both with natural teeth only as well as with natural plus replaced teeth. However, that study reported on an elderly population (55 years and older) and did not distinguish between fixed (FDP) and removable dental prostheses (RDP). Moreover, although number of teeth and number of occluding anterior and posterior teeth were used as a measure for dental status, the actual configurations of the dentitions involved remains unclear.

Several authors have made attempts to classify dentitions according to the number of teeth, tooth location, or number of occluding pairs. For example, the Eichner index has been validated for an elderly Japanese population with respect to chewing ability [17]. However, the Eichner index does not specify the number and location of teeth present and is therefore not reflecting oral functionality in detail, for example aesthetics. According to a systematic review sufficient oral functional depends on the presence of minimally 20 teeth with nine to 10 occluding pairs, no tooth loss in the anterior region, and the retention of premolars, whereas there may be little increase in satisfaction seen in subjects with presence of molars [18]. Recently, a hierarchical classification system has been described that reflects oral functionality according to the conclusions of this review [19, 20]. In this classification system, oral functionality is expressed by (1) number of teeth in upper and lower jaw, (2) completeness of anterior regions, (3) number of premolar occluding pairs, and (4) number of molar occluding pairs. This classification system has not yet been used to assess chewing ability in relation to dental status in China.

The aim of this study was to assess the relationship among dental conditions according to the proposed dental functional classification system (without and with FDP or RPD) and chewing ability of Chinese subjects of 40 years and older. It was hypothesized that subjects with FDP will have more benefit from added artificial teeth with respect to chewing ability than subjects with RDP.

## Materials and methods

The study was conducted in the Qingdao area, located at the east coast of Shandong Province, the latter situated in Eastern China. Shandong is one of the largest provinces in China in terms of population (94 million in 2008) and economy. Qingdao City has approximately 3 million inhabitants. Qingdao City has direct jurisdiction over the surrounding rural territory in Shandong Province, including five county-level cities (approximately 200.000–400.000 residents each) and the counties surrounding these cities. Each rural county comprises 40–80 small rural villages. The total area of Qingdao (urban and rural) is approximately 10,000 km^2^ with a coastline of approximately 730 km and has approximately 8 million inhabitants.

### Sampling method

For this study, a cross-sectional survey, representing 1,588 subjects aged ≥40 years living in urban and rural areas in Qingdao, Shandong Province, was conducted. To calculate the sample size needed, it was decided that the sample should allow for multivariable logistic regression with at least 12 independent variables among dentate subjects. This implies that at least 120 observations of the least prevalent part of a dichotomous variable among dentate subjects are necessary. Using 8 % prevalence as a worst-case scenario, a total sample size of 1,500 is needed to attain the 120 observations needed. To allow for an estimated 5 % prevalence of edentulous subjects, the targeted population was increased to 1,575.

Subjects were selected randomly from administrative lists of residents of communities or villages provided by local authorities and lists of employees of factories. Inclusion aimed at proportional distribution according to age categories, gender, and place of residence (urban or rural). Data were collected in 2009 and 2010.

The urban sample was constructed after consulting local authorities on the basis of accessibility, and comprised 11 communities and four factories in Qingdao City. Administrators of the communities informed and invited their residents for participation in this study. The examination venue usually was a neighborhood community office or a social center for the elderly. A total of 570 community inhabitants and 193 employees from factories were included on the basis of voluntary participation. As truly representative sampling was not feasible the pathfinder sampling method was adopted incorporating sufficient examination sites to cover relevant groups of the population intended [21]. It appeared that subjects of certain age categories were underrepresented in the initial urban sample (mostly males). Therefore, a complementary convenient sub-sample, drawn from community residents attending a health centre while they were waiting for a periodical check-up, was eventually included. Fifty-three subjects were included in this way.

For the rural sample, one county (Zhugou) considered representative for northeast Shandong Province was chosen on the basis of accessibility for investigating dental health status and cooperation from local authorities. This county (a predominantly agrarian area with a low population density and a total population of approximately 36,000) is located approximately 120 km northwest from Qingdao City and comprises 56 villages ranging from 153 to 1,583 inhabitants. On the basis of information from the local authorities, it appeared that there were large differences in income among the villages. As gross domestic product (GDP) was expected to be related with socio-economic status (SES), 10 villages with different GDP were selected randomly: three villages out of 19 with highest 2008 GDP; four out of 18 with middle GDP, and three out of 19 with lowest GDP. Next, subjects from these villages were randomly selected using administrative name-lists. In cases where subjects were invited but did not show up (*n* = 347, 45 %), other subjects were randomly drawn from the same sampling lists.

The research was carried out in compliance with the Helsinki Declaration and was approved by the ethics committee of the medical school at Qingdao University, Qingdao, China.

### Participants

Of the 1,588 subjects participating in the epidemiological study, 126 subjects (8 %) were edentulous in one or both jaws and were excluded from the present analyses. The remaining 1,462 subjects dentate in upper and lower jaw were included (Table 1). A previous report of this survey revealed that of all dentate subjects, 59 % (*n* = 861) had a natural dentition without any tooth replacement, 30 % had an FDP (*n* = 441), and 11 % (*n* = 160) had an RDP. Forty-three subjects (3 % of the total sample) had both FDP and RDP. The majority of subjects with FDP (57 %) had one or two teeth replaced; the majority of RDPs replaced three or more teeth (78 %) [20]. More details with respect to number of teeth and tooth replacements have been described in that report.

**Table 1:** Number (%) of included subjects (*n* = 1,462) dentate in upper and lower jaw according to gender and place of residence, distribution of SES, age (minimum, maximum, and mean), and OHIP-14CN total score (minimum, maximum, and mean)

	Urban	Rural	Total
Female	405 (58)	297 (42)	702 (48)
Male	385 (51)	375 (49)	760 (52)
Total	790 (54)	672 (46)	1,462 (100)
	SES high	SES middle	SES low
SES^a^	583	449	428
	Minimum	Maximum	Mean (SD)
Age	39	87	54.95
OHIP-14CN	0	54	7.76

^a^SES data of two subjects missing

### Questionnaire

Subjects were asked to complete a structured questionnaire that was used previously in a study in Vietnam [19] and translated into Mandarin. The Chinese version was checked for language adequacy by a panel of dentists and pilot tested on 20 Chinese subjects to assess clarity. Some minor modifications were made based on the results of the pilot. The questionnaire included the Chinese short version of the Oral Health Impact Profile (OHIP-14CN) [22], demographic information (age, gender, and place of residence), SES (modified Kuppuswami’s SES classification [23]), and questions that asked whether the subject was able to chew eight different foods common for Chinese people. The eight foods were listed randomly in the questionnaire and included four foods that Chinese people consider as soft (cooked rice, steamed bread, Shaobing (Chinese style baked roll), meat) and four that are considered as hard (raw vegetables, raw carrots, apples, and nuts). Perceived difficulty of chewing was scored as: 1 = very easy to chew; 2 = minor problems with chewing, got used to it; 3 = minor problems, cannot get used to it; 4 = difficult to chew, not avoiding this food; 5 = very difficult to chew, not avoiding; 6 = very difficult to chew, avoiding this food; 7 = not avoiding this food, never eating it. OHIP-14CN was included in the questionnaire to assess OHRQoL. Responses on each OHIP question were given on a 5-point Likert scale (0 = never, 1 = hardly ever, 2 = occasionally, 3 = fairly often, 4 = very often) for a reference period of 3 months.

Subjects not able to complete the questionnaire themselves (e.g., because of illiteracy or visual impairment) were helped by an assistant who read aloud the questions and recorded the answers. After completion, the questionnaire was checked for unrecorded items, and if applicable, subjects were requested to complete the form.

### Clinical examination

After obtaining verbal consent from the participants, a clinical examination was conducted by a calibrated examiner following the procedures and diagnostic criteria recommended by the World Health Organization [24]. Inter-observer agreements between the principal investigator and experienced researchers in the field on DMFT variables were excellent (kappa’s ≥ 0.89). Of all variables recorded, only the presence of teeth (including third molars), tooth type, number and location of natural POPs, and tooth replacements were considered in the present study. Roots were considered non-functional teeth with respect to chewing ability, and therefore considered as missing teeth. A natural POP was considered as a posterior occluding pair of natural teeth. A distinction was made between teeth replaced by FDP and those replaced by RDP. A replaced tooth was defined as a missing tooth replaced by FDP or RDP. Mean numbers of POPs added by FDP or RDP were also considered.

### Dental functional status classification system

In the classification system [19], dentitions were classified on the basis of a dichotomized five level branching hierarchy in which the criteria applied on the levels are based on conditions that reflect functionality (Table 2). With regard to each level in the branching hierarchy, the number of natural teeth, the tooth types present, and the number of natural POPs were calculated. Subjects were classified in two ways. First, subjects were classified on the basis of their configuration of natural teeth only (Class_nat_). Next they were reclassified on the basis of configurations including natural teeth plus teeth replaced by FDP (Class_F_) and/or RDP (Class_R_) [20].

**Table 2:** Levels and criteria for dichotomization of the step-by-step branching hierarchy used and percentages of subjects (*n* = 1,462) classified in the subsequent categories based on natural teeth only (Class_nat_)

Level	Meeting criterion		Dichotomy
	Yes	No	
I Dentition level	≥1 Tooth present in each jaw 100 %		≥1 Tooth vs. no teeth
II Jaw level	≥10 Teeth in both upper *and* lower jaw 82 %	<10 Teeth in upper *or* lower jaw 18 %	≥10 Teeth vs. <10 teeth
III Anterior level	All 12 anterior teeth present 72 %	<12 Anterior teeth 28 %	Complete vs. incomplete
IV Premolar level	3 or 4 Occluding pairs of premolars 75 %	≤2 Occluding pairs of premolars 25 %	‘Sufficient’ vs. ‘impaired’
V Molar level	≥1 Occluding pairs of molars at both left *and* right side of the dentition 74 %	No occluding pairs of molars at left *or* right side of the dentition 26 %	‘Sufficient’ vs. ‘impaired’

### Data analyses

Two approaches were used to analyze chewing ability in relation to dental conditions. First, the relationships were analyzed for the conditions of the different dental regions separately. In the second approach, chewing ability was related to the hierarchical functional classification system, in which the dental regions are considered in the context of the conditions of the dentition as a whole.

In the first approach—in which the relationship between chewing ability and the separate dental regions is analyzed—multivariable logistic regression models were used. In these models ‘chewing problems’ was the dependent variable; the conditions at the levels II to V (≥10 teeth in each jaw, anterior region complete, premolar region sufficient, and molar region sufficient) and tooth replacement were the independent variables. Possible associations between the condition of the separate dental regions and chewing problems were adjusted for a number of background variables. The following background variables were included in the models: OHIP-14CN total score (dichotomized using the sample median as the cut-off point); questionnaire administration (completely self-administered vs. (partly) assisted by a dental assistant); the demographic variables age (age categories 40–49, 50–59, 60–69, and 70 years and older), gender, and place of residence (urban or rural); and socio-economic status (three levels).

With respect to chewing ability of the eight separate foods, the answers ‘very easy to chew’, ‘minor problems with chewing, got used to it’, and ‘minor problems, cannot get used to it’ were considered ‘no or minor problems with chewing’; the answers ‘difficult to chew, not avoiding this food’, ‘very difficult to chew, not avoiding’, and ‘very difficult to chew, avoid this food’ were considered ‘chewing problems’. In the analysis of chewing ability of the respective foods, outcomes were dichotomized as follows: ‘no or minor chewing problems’ (scores 1,2, and 3) vs. ‘chewing problems’ (scores 4, 5, and 6). Score 7 (‘not avoiding this food, never eating it’) was considered missing.

Combined soft and hard foods were analyzed at a different level in which a more stringent criterion was applied. Cut-off for dichotomization here was defined: ‘no chewing problems’ (score 1 (‘very easy to chew’) for each of the four combined foods) vs. ‘chewing problems’ (score >1 for at least one of the combined foods).

The performance of the multivariable logistic models was expressed as the percentages of subjects having chewing problems predicted by (1) the dental conditions only and (2) all variables. To express the performance of the logistic models, the area under the curve (AUC) statistic is used. An AUC of 0.5 indicates a total absence of model fit; an AUC of 1 belongs to a situation where model fit is perfect. Although the models are etiologic by nature and not meant as a predictive tool, the percentage predicted correctly are presented as an additional indication of the model fit.

In the second approach—in which the relationship between chewing ability and dental functional status is analyzed—likelihood ratios were calculated after dichotomization (meeting vs. not meeting the respective dental conditions). These likelihood ratios express the extent to which a given condition, for instance having at least 10 teeth in each jaw, discriminates between people with and without chewing problems with soft (Ls) and hard foods (Lh), respectively. A likelihood ratio of 1 indicates a classification criterion that is not discriminatory.

Both approaches were applied to the dental conditions based on (1) natural teeth only (Class_nat_) and subsequently (2) on levels based on natural teeth plus teeth replaced by FDP (Class_F_) and/or RDP (Class_R_).

R software version 2.13.1 was used for the statistical analyses [25].

## Results

### Chewing ability and dental conditions based on natural teeth only

**Fig. 1:**
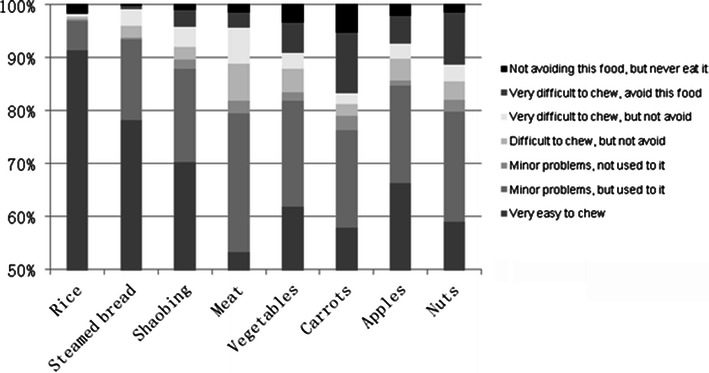
Percentage distribution of subjects dentate in each jaw (*n* = 1,462) reporting ease or various difficulties with chewing the eight foods investigated

The majority of subjects (78–91 %) reported no or minor problems with chewing of the foods (Fig. 1). More problems, in terms of frequency as well as severity, were reported for hard foods than for soft foods except for meat. The percentage of subjects reporting ‘easy to chew’ was lowest for meat (53 %) and highest for cooked rice (91 %), whereas ‘very difficult to chew, but not avoiding this food’ was lowest for cooked rice (0.5 %) and highest for meat (7 %). The percentage of subjects avoiding foods because of difficulties with chewing was low (<5 %) except for carrots (12 %) and nuts (10 %).

Having ‘sufficient’ molar region compared to not meeting this criterion reduced the chance for having chewing problems significantly for all foods with a factor 0.47 (Table 3: OR for Shaobing, 0.53) to 0.67 (OR for carrots, 0.33). Having ‘sufficient’ premolar region reduced the chance for having chewing problems with a factor 0.42 to 0.53 for all foods except for cooked rice and steamed bread. Incomplete anterior regions was only associated with problems about chewing apples (OR = 0.64). On the basis of dental conditions only, the percentages of correctly predicted subjects having chewing problems ranged from 65.6 (for meat) to 85.8 % (for cooked rice); AUCs ranged from 0.673 to 0.715, showing a reasonably high level of predictability of the model. The full model, including all 10 variables, predicted 73.8 (again for meat) to 93.5 % (again for cooked rice) of the subjects having chewing problems; AUCs ranged from 0.803 to 0.844.

**Table 3:** Odds ratios [95 % CI] for having ‘chewing problems’ with chewing for the eight foods according to the dental condition in the multivariable logistic regression model, adjusted for the background variables OHIP-14CN total score, age, gender, place of residence, SES, and questionnaire administration format

Condition^a^ (level)	OR [95 % CI]							
	Rice	Steamed bread	Shaobing	Meat	Vegetables	Carrots	Apples	Nuts
≥10 Teeth in each jaw (II)	1.17	0.60	0.70	1.14	0.89	0.94	0.89	0.83
	[0.50–2.74]	[0.35–1.04]	[0.42–1.18]	[0.68–1.92]	[0.53–1.48]	[0.55–1.60]	[0.53–1.50]	[0.49–1.40]
Anterior regions complete (III)	0.65	0.87	0.96	0.73	0.79	0.78	**0.64**	0.67
	[0.38–1.13]	[0.59–1.27]	[0.68–1.36]	[0.53–1.00]	[0.57–1.09]	[0.56–1.08]	[0.46–0.89]	[0.48–0.93]
Premolar region ‘sufficient’ (IV)	0.96	0.66	**0.57**	**0.48**	**0.58**	**0.56**	**0.50**	**0.47**
	[0.48–1.92]	[0.43–1.02]	[0.39–0.84]	[0.33–0.70]	[0.40–0.85]	[0.38–0.83]	[0.34–0.73]	[0.32–0.69]
Molar region ‘sufficient’ (V)	**0.46**	**0.44**	**0.53**	**0.48**	**0.46**	**0.33**	**0.46**	**0.45**
	[0.26–0.80]	[0.29–0.65]	[0.37–0.77]	[0.34–0.68]	[0.33–0.65]	[0.24–0.47]	[0.32–0.66]	[0.32–0.65]
	
AUC dental conditions	0.682	0.715	0.693	0.673	0.676	0.704	0.705	0.698
Percentage correctly predicted by dental conditions only	85.8	79.5	74.0	65.6	69.8	70.6	73.2	67.9
AUC dental conditions plus background variables	0.821	0.844	0.829	0.808	0.803	0.816	0.834	0.830
Percentage correctly predicted by dental conditions plus background variables	93.5	84.1	79.0	73.8	75.9	75.8	78.8	76.3

Bold figures indicate significant relationships. Functional levels based on natural teeth only (Class_nat_)

*AUC* area under curve

^a^Reference = condition not present

The multivariable logistic regression analysis for combined soft and combined hard foods revealed that subjects with the condition ‘sufficient premolar region’ and subjects with the condition ‘sufficient molar region’ had significantly less chance to have chewing problems compared to subjects not meeting the dental conditions (Table 4). The condition ‘anterior regions complete’ showed significantly less chewing problems for hard foods. On the basis of the dental conditions only, the percentage of correctly predicted subjects having chewing problems was 65.0 % for soft foods and 64.1 % for hard foods (both AUCs 0.670). The full model predicted 73.8 % for soft foods and 74.4 % for hard foods correctly (AUCs 0.805 and 0.808, respectively).

**Table 4:** Odds ratios [95 % CI] for having ‘chewing problems’ for the combined soft (cooked rice, steamed bread, Shaobing, and meat) and combined hard (raw vegetables, raw carrots, apples, and nuts) foods according to the dental condition in the multivariable logistic regression model, adjusted for the background variables OHIP-14CN total score, age, gender, place of residence, SES, and questionnaire administration format

Condition^a^ (level)	Soft foods		Hard foods	
	OR	95 % CI	OR	95 % CI
≥10 Teeth in each jaw (II)	1.10	[0.65–1.84]	0.78	[0.46–1.33]
Anterior regions complete (III)	0.76	[0.55–1.05]	**0.68**	[0.50–0.95]
Premolar region ‘sufficient’ (IV)	**0.54**	[0.37–0.78]	**0.63**	[0.43–0.92]
Molar region ‘sufficient’ (V)	**0.44**	[0.31–0.62]	**0.41**	[0.29–0.59]
	
AUC dental conditions	0.670		0.670	
Percentage correctly predicted by dental conditions only	65.0		64.1	
AUC dental conditions plus background variables	0.805		0.808	
Percentage correctly predicted by dental conditions plus background variables	73.8		74.4	

Bold figures indicate significant relationships. Functional levels based on natural teeth only (Class_nat_)

*AUC* area under curve

^a^Reference = condition not present

**Fig. 2:**
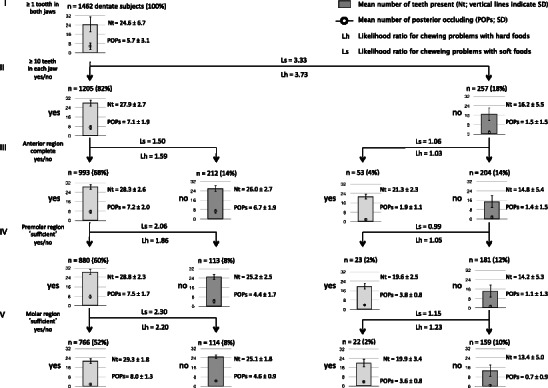
Distribution of subjects dentate in each jaw (*n* = 1,462) according to the functional classification system [19] and likelihood ratios for having problems with chewing: *I* dentate in each jaw, *II* ≥10 natural teeth in each jaw, *III* anterior region complete, *IV* premolar region ‘sufficient’, *V* molar region ‘sufficient’. *Dark columns* indicate status of not meeting the criterion

The branching hierarchy shown in Fig. 2 describes 82 % of all subjects dentate in each jaw (*n* = 1462) up to level IV (premolar region) and 72 % up to level V (molar region). Categories not meeting the dental conditions in the ‘≥10 teeth in each jaw’ branch and categories meeting the dental conditions in the ‘<10 teeth in each jaw’ branch were not further dichotomized to the next level. At level II of the classification system (‘≥10 teeth in each jaw’) the likelihood ratio for chewing problems with combined soft foods (Ls) was 3.33. The likelihood ratio with hard foods (Lh) at this level was 3.74. In the branch of ‘≥10 teeth in each jaw’, subjects with incomplete anterior region had a higher chance for having chewing problems (level III: Ls = 1.50; Lh = 1.59). In this branch likelihood ratios for the premolar regions (level IV) were: Ls = 2.06 and Lh = 1.86; for the molar region (level V) they were Ls = 2.30 and Lh = 2.20, respectively. In the branch of ‘<10 teeth in each jaw’, likelihood ratios for having chewing problems ranged from 0.99 to 1.06 for the anterior and premolar regions. For the molar region, Ls and Lh were 1.15 and 1.23, respectively.

Multivariable logistic analysis in which the variable ‘tooth replacement’ was added in the model based on natural teeth (Class_nat_) revealed that tooth replacement was clearly not significantly related with the chance for having chewing problems. In all four models (two models for soft foods with FDP and/or RDP and two for hard foods), the *p* value for this variable was larger than 0.5 (not in Tables).

### Chewing ability and dental conditions based on natural teeth plus teeth replaced by FDP or RDP

After reclassification to categories based on natural teeth plus tooth replacements (Class_F_ and/or Class_R_) subjects with FDP had more chewing problems with hard foods than their counterparts with natural teeth only: OR = 1.45 (*p* = 0.009; Table 5). Subjects with RDP had more chewing problems than their counterparts with natural teeth only: OR = 1.96 (*p* < 0.001) for soft foods and OR = 2.20 (*p* < 0.001) for hard foods. In this model, the condition ‘sufficient premolar region’ as well as the ‘sufficient molar region’ were significant for all foods. On the basis of the dental conditions only, the percentage of correctly predicted subjects with chewing problems was between 63.7 and 65.1 %. The full model predicted 73.6–74.5 % of subject with chewing problems correctly.

**Table 5:** Odds-ratios, *p* values, and 95 % confidence intervals (CI) of the multivariable logistic regression analysis for having chewing problems with combined soft and combined hard foods with dental status after reclassification to Class_F_ and Class_R_, adjusted for the background variables OHIP-14CN total score, age, gender, place of residence, SES, and questionnaire administration format

Condition^a^ (level)	Soft foods						Hard foods					
	In class_F_			In class_R_			In class_F_			In class_R_		
	OR	*P*	95 % CI	OR	*P*	95 % CI	OR	*P*	95 % CI	OR	*P*	95 % CI
≥10 teeth in each jaw (II)	1.22	0.516	0.67–2.23	1.36	0.294	0.77–2.40	1.12	0.713	0.60–2.09	0.84	0.565	0.47–1.51
Anterior regions complete (III)	0.83	0.279	0.58–1.17	**0.69**	0.028	0.50–0.96	**0.65**	0.015	0.45–0.92	**0.64**	0.008	0.46–0.89
Premolar region ‘sufficient’ (IV)	**0.60**	0.017	0.39–0.91	**0.50**	<0.001	0.34–0.74	**0.58**	0.013	0.38–0.89	**0.62**	0.015	0.42–0.91
Molar region ‘sufficient’ (V)	**0.38**	<0.001	0.26–0.56	**0.41**	<0.001	0.29–0.59	**0.32**	<0.001	0.22–0.48	**0.42**	<0.001	0.29–0.60
Teeth replaced	1.29	0.071	0.98–1.70	**1.96**	<0.001	1.31–2.94	**1.45**	0.009	1.10–1.92	**2.20**	<0.001	1.45–3.33
	
AUC dental conditions	0.670			0.672			0.683			0.671		
Percentage subjects correctly predicted by dental conditions only	63.7			65.1			64.4			64.3		
AUC dental conditions plus background variables	0.804			0.806			0.813			0.806		
Percentage subjects correctly predicted by dental conditions plus background variable	73.9			73.6			74.5			74.0		

Bold figures indicate significant relationships

*AUC* area under curves

^a^Reference = condition not present

In general, likelihood ratios for having chewing problems for subjects classified on the basis of natural teeth plus replaced teeth (Class_F_/Class_R_) were higher than likelihoods for subjects classified on the basis of natural teeth only (Class_nat_), except for ‘premolar region impaired‘ and ‘molar region impaired’ for both soft and hard foods (Table 6). Likelihood ratios for having chewing problems were highest at the level of ‘≥10 teeth in each jaw’ (Ls = 3.33 in Class_nat_ to Lh = 5.12 in Class_F_/Class_R_). For the subsequent predictors, likelihood ratios for having chewing problems were generally higher if the conditions at proceeding levels were met than if the conditions at preceding levels were not met. For instance, the predictor ‘anterior region incomplete’ revealed likelihood ratios for having chewing problems of 1.50 and 1.55, respectively, for soft foods if the subjects had ‘≥10 teeth in each jaw’; when subjects did not meet the condition ‘≥10 teeth in each jaw’ likelihood ratios were 1.06 and 1.36, respectively.

**Table 6:** Likelihood ratios for having chewing problems with combined soft and hard foods according to the condition of meeting/not meeting a functional level in the hierarchical classification system at the cut-off for the next level, based on natural teeth only (Class_nat_) and on natural teeth plus replaced teeth (Class_F_/Class_R_)

Condition			Predictor	Soft foods		Hard foods	
≥10 teeth in each jaw	Anterior region complete	Premolar region ‘sufficient’		Class_nat_	Class_F_/class_R_	Class_nat_	Class_F_/class_R_
			<10 teeth in each jaw	3.33 (62)	4.14 (23)	3.73 (53)	5.12 (18)
Yes			Anterior region incomplete	1.50 (100)	1.55 (78)	1.59 (93)	1.68 (71)
No			Anterior region incomplete	1.06 (15)	1.36 (10)	1.03 (12)	1.75 (8)
Yes	Yes		Premolar region ‘impaired’	2.06 (47)	1.96 (34)	1.86 (48)	1.83 (34)
No	No		Premolar region ‘impaired’	0.99 (5)	1.10 (2)	1.05 (6)	1.24 (2)
Yes	Yes	Yes	Molar region ‘impaired’	2.30 (47)	2.74 (34)	2.20 (46)	2.43 (35)
No	No	No	Molar region ‘impaired’	1.15 (9)	1.33 (1)	1.23 (9)	0.87 (0)

*n* smallest number in the four cells in the respective comparisons

## Discussion

This study aimed to investigate chewing ability in Chinese adults without and with prosthodontic replacements. As this study is part of a larger epidemiological survey, sample construction aimed at proportional distribution of subjects according to place of residence, gender, and age categories. With the aid of the local governmental administrative system this goal was reasonably well met in the rural area; therefore, the rural sample is considered to reflect the rural population in Shandong Province. In the urban area inclusion of the intended subjects through administrative lists appeared to be more complicated. To deal with this, a pathfinder sampling method was used to find subjects from randomly chosen communities and factories. Eventually, unfilled cells were filled with community residents attending a health center for periodical check-up. Although the composition of this convenient sub-sample (which is 6 % of the total urban sample) was slightly different from the total urban sample with respect to SES and gender (i.e., males above the age of 70 were not represented in the sub-sample), we consider that the urban sample reflects the population of Qingdao City.

The hierarchical dental functional classification system applied in the present study has been previously evaluated [19, 20]. The homogeneities of the dichotomized groups appeared to be satisfactory; we therefore consider the classification system as appropriate to describe the dental functional status of a population.

The Chinese diet, unlike western diets, contains few hard fibrous foods and most foods frequently eaten by Chinese people are steamed, fried, or boiled [16]. For this reason we included uncooked foods in the questionnaire, which were assigned to the category ‘hard foods’. Depending on its preparation, meat could be considered soft or hard as reflected in the outcomes of this study.

Although the majority of subjects reported no or minor chewing problems, this study demonstrated a significant association between dental functional status and chewing difficulties with the foods. The present study did not address possible associations between chewing ability and temporo-mandibular disorders (TMD). However, reported difficulties with chewing might include TMD symptoms. It has been suggested that chewing ability is correlated with dysfunction of TMD patients [26]. The present paper is focusing on the relationship between chewing ability and dental (occlusal) conditions. Several studies and reviews reported lack of evidence with respect to associations between occlusal factors—such as missing posterior teeth—and TMD problems [27–30]. In contrast, some studies associated higher risk for TMD problems with dentitions with asymmetric occlusal support [31, 32]. Based on these considerations it was expected that TMD patients would be more or less equally distributed among the subjects as categorized by the ‘occlusal’ classification system and thus not biasing the results.

Multivariable logistic regression analyses showed that the condition ‘sufficient molar region’ based on natural teeth only (Class_nat_) significantly reduced the chance to have chewing problems with all soft and hard foods as compared to an impaired molar region. The condition of the premolar region appeared to be not relevant for the two softest foods (cooked rice and steamed bread) but affected chewing ability for the other foods. The importance of sufficient POPs in premolar and molar regions for chewing ability has been demonstrated in other studies, both for natural and artificial POPs [3, 16, 33]. Moreover, it has been suggested that impaired molar areas cause more chewing problems than impaired premolar areas [33], which is in accordance with the present result showing the importance of molars when considered as an isolated dental condition.

The condition ‘complete anterior region’ did not significantly affect chewing ability, with the exception of ‘chewing’ apples; obviously because anterior teeth are needed for biting off apples. Notably, the condition ‘≥10 teeth in each jaw’ was not associated with chewing problems when considered as an isolated condition. However, the odds ratios in the multivariable regression analyses—in which the conditions at the separate levels of the dental functional system *sec* are the independent variables—only describe the chance for having chewing problems related to that specific (isolated) dental condition. Apparently having at least 10 teeth in each jaw does not affect chewing ability if other dental conditions such as complete anterior region or sufficient premolar region are not taken into account. In other words, expressing the dental status only by having more or less than 10 teeth in each jaw did not discriminate with respect to chewing problems.

In contrast, when subjects’ dentitions are classified hierarchically within the frame of the classification system and taking into account the other dental conditions, the condition ‘≥10 teeth in each jaw’ was highly correlated with having chewing problems (Fig. 2; likelihood ratios ranged from 3.33 to 3.74). If the conditions ‘≥10 teeth in each jaw’ and ‘anterior region complete’ were met, ‘sufficient premolar region’ and ‘sufficient molar region’ were relevant to reduce the likelihood ratios for chewing problems (both in the order of a factor 2), both for soft and for hard foods. This reduction of likelihood ratios with approximately a factor 2 might be related with the ‘reduction’ of POPs: from seven to eight POPs when the conditions were met, to four or five when the conditions were not met (see Fig. 2). Although the level of evidence for the shortened dental arch concept has been appraised differently [34, 35], a review indicated that reduced dentitions without molar posterior support could provide sufficient oral function but that complaints on perceived oral function may arise, especially for hard foods [34]. The findings of the present study showed that the presence of bilaterally at least one molar POP was relevant for chewing ability, but surprisingly not for hard foods only but also for soft foods. With respect to perceived chewing ability we expected discriminatory differences between soft and hard foods. However, overall differences in outcomes between soft and hard foods (ORs in Tables 3, 4, 5, and 6 and likelihood ratios in Fig. 2) were relatively small.

In the hierarchy branch for the condition ‘<10 teeth in each jaw’, the subsequent dental conditions hardly affected chewing ability. This could be expected as less than 10 teeth in each jaw means a large variation in ‘deficiencies’, as is demonstrated by the large variation (SDs) of POPs in this branch.

If missing teeth were replaced by FDP or RDP, subjects had similar chance for chewing problems as their counterparts with similar dental conditions without these replacements. In other words, the natural teeth present predominantly determined chewing ability for the eight foods, and not the artificial teeth added. This indicates that the group of subjects with tooth replacement cannot be considered as ‘complainers’ with respect to chewing.

However, when comparing chewing ability in dentitions with natural plus artificial teeth, artificial teeth seem to perform less (more chewing problems) than natural teeth. The hypothesis that subjects with FDP have more benefit from added teeth than subjects with RDP has to be accepted for soft foods only. This finding is in line with findings from a randomized controlled clinical trial, in which patients with shortened dental arches extended with FDP were more satisfied than patients with RDP [36]. Also it was suggested that RDPs add little in avoiding chewing problems [3]. The wide confidence intervals found for the odds ratios (Table 5) are most probably due to the wide variations in numbers of teeth replaced by RPD. In this respect, it is worthwhile to mention that a recent review on chewing function reported that an association between the change in objective chewing capacity and self-assessed chewing ability as a result of tooth replacements has not been demonstrated so far [37].

However, also the quality of the tooth replacements, including the indications for FDP and RDP might play a role in this matter. It has been stated before that, especially in rural areas in China, dental care providers (which are not necessarily dentists) prefer to extract affected teeth above treatments that aim at the retention of such teeth. They seem to practice rather unconventional prosthodontic principles, in which they tend to provide FDPs for low prices rather than RDPs, even when only very few teeth are available as abutment teeth [38]. Indeed, FDPs were more often found among rural subjects than among urban subjects, whereas the reverse tendency was seen for RDP [39].

The present study demonstrated that the hierarchical functional classification system was appropriate to predict subjects with chewing problems to a considerable extent.

## Conclusions

- A minority of a Chinese adult population in Shandong Province reported serious chewing problems.
- The condition ‘≥10 teeth in each jaw’ appeared to have high impact on chewing ability; the conditions ‘sufficient premolars region’ and ‘sufficient molar region’ contributed equally to chewing ability.
- In this population artificial teeth added by FDP and RDP performed less than natural teeth.
